# Preparation of hollow iron/halloysite nanocomposites with enhanced electromagnetic performances

**DOI:** 10.1098/rsos.171657

**Published:** 2018-01-24

**Authors:** Xue-Gang Chen, Ru-Chang Li, Ao-Bo Zhang, Shuang-Shuang Lyu, Shu-Ting Liu, Kang-Kang Yan, Wei Duan, Ying Ye

**Affiliations:** 1 Ocean College, Zhejiang University, Zhoushan 316021, People's Republic of China; 2 Zhejiang Institute of Geological Survey, Hangzhou 310007, People's Republic of China; 3 Zhejiang Institute of Geology and Mineral Resources, Hangzhou 310007, People's Republic of China; 4 College of Earth Sciences, Chengdu University of Technology, Chengdu 610059, People's Republic of China

**Keywords:** halloysite, microwave absorption, magnetic performance, permeability, nanotubular structure

## Abstract

Nanostructures loaded on halloysite nanotubes (HNTs) have attracted global interest, because the nanotubular HNTs could extend the range of their potential applications. In this study, we fabricated a novel nanocomposite with hollow iron nanoparticles loaded on the surface of HNTs. The structure of the iron nanoparticles can be adjusted by ageing time. Owing to the increased remnant magnetization and coercivity values, the nanocomposites loaded with hollow iron nanoparticles showed better electromagnetic performance than that with solid iron nanoparticles. This study opens a new pathway to fabricate halloysite nanotubular nanocomposites that may gain applications in the catalytic degradation of organic pollutants and electromagnetic wave absorption.

## 1. Introduction

Nanotubular materials have potential applications in catalysis, magnetic field, wastewater treatment, polymer filler and microwave absorption due to their advantages of low-density, high surface area, high strength and nanoencapsulation [1–3]. Halloysite is a natural aluminosilicate similar to kaolinite [4,5]. Halloysite was conventionally used to produce porcelain [6] or as a filler for polymers [7–9]. On the other hand, its natural nanotubular structure makes it a competitive substitute for carbon nanotubes [10]. The performance of functional materials loaded on halloysite nanotubes (HNTs) will be enhanced and their potential applications could be extended. In addition, using natural halloysite as a raw material could enlighten the economic utilization of naturally occurring and abundantly available minerals. Therefore, various functional materials have been loaded into/onto the HNTs, such as TiO_2_ [11,12], ferrite [2,13,14], catalysts [15–17] and drugs for controllable release and delivery [18–21]. Iron and iron alloys are important functional materials that can be used in various fields. For instance, both Fe and Fe-alloys (such as FeNi and FeCo) are excellent microwave absorbers due to their high magnetic loss (natural resonance, eddy current effect or hysteresis loss) for incident microwaves [22–25]. Ascribed to their high surface activity, they also show potential applications in wastewater treatment, e.g. rapid catalytic degradation of organic contaminants [26–28]. In addition, previous studies suggested that materials with a hollow interior exhibited not only improved catalytic activity and magnetic performance, but also significantly decreased densities [29,30].

## 2. Results and discussion

**Figure 1.**
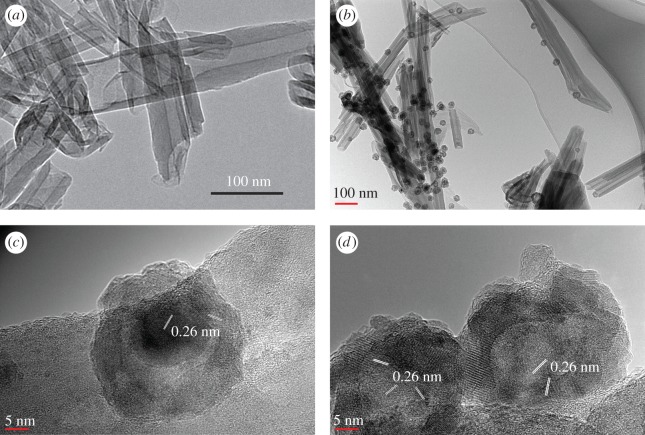
TEM images of (*a*) natural halloysite, (*b*) hollow iron/halloysite nanocomposite prepared with an ageing time of 1 h (HY1), HRTEM images of (*c*) a core/shell iron nanoparticle and (*d*) hollow iron nanoparticles attached onto the HNT.

In this study, we prepared a novel hollow iron/halloysite nanocomposite (HIH) using metal oleate as a precursor [2,31] (electronic supplementary material). As shown in figure 1, the original halloysite presents nanotubular structures with an inner diameter of 10–25 nm and a shell thickness of 8–25 nm. When loaded with iron nanoparticles, the original morphology of HNTs was largely preserved. The inner diameter and the shell thickness were maintained at approximately 20 nm, indicating that calcination at 450°C during preparation did not significantly destroy the structure of HNTs. Iron nanoparticles with a diameter of 20–50 nm and an inner diameter of 10–30 nm dispersed on the outer surface of HNTs. The loading of the nanoparticles was relatively low due to the low dosage of Fe^2+^-oleate. HRTEM images (figure 1*c*,*d*) indicated that the prepared iron nanoparticles mainly include hollow nanoparticles and core/shell nanoparticles. Nevertheless, the core, shell and the hollow shell all show a basal spacing of 0.26 nm, which may be ascribed to the fact that the iron nanoparticles have been partly surface oxidized to Fe_2_O_3_. The observed basal spacing of 0.26 nm was ascribed to the (311) crystal faces of Fe_2_O_3_ [32,33].

**Figure 2.**
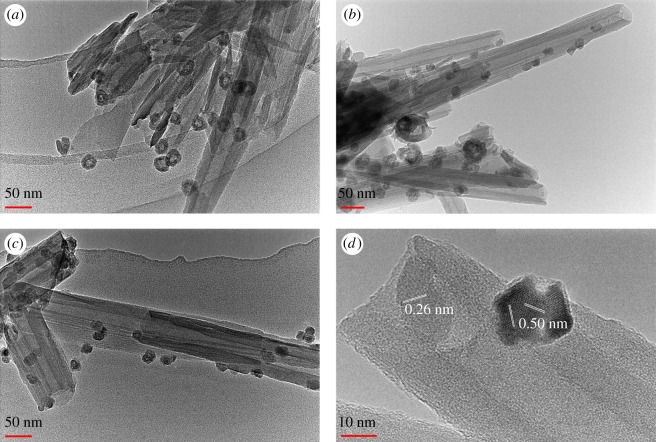
TEM images of hollow iron/halloysite nanocomposites prepared at different ageing times (*a*) 4 h, (*b*) 8 h, (*c*) 24 h and (*d*) HRTEM image of a solid iron nanoparticle attached on the surface of (*c*).

The structure of the iron nanoparticles can be adjusted by the ageing time. As shown in figure 2, with the increase of ageing time from 4 to 24 h, the structure of the nanoparticles changed gradually from hollow (figure 2*a*) to a mixing of hollow and solid particles (figure 2*b*), and finally to all solid particles (figure 2*c*). The initial morphology of HNTs was preserved during preparation. A HRTEM image on the solid nanoparticles suggests that the basal spacings are 0.26 and 0.50 nm, respectively, corresponding to the (311) crystal faces of Fe_2_O_3_. According to previous studies, the formation of hollow iron nanoparticles may be attributed to the presence of residual sodium oleate in the Fe^2+^-oleate complex via an effect called ‘molten salt corrosion’ [33]. The Kirkendall effect may also play a role [34,35] that the hollow structure was formed during the transformation of Fe^2+^-oleate complex to iron oxides, and finally to iron. Probably due to the Kirkendall effect declining with increasing ageing time, solid iron nanoparticles predominate at ageing time of greater than 8 h.

The crystal structures of HNTs and HIHs were investigated by X-ray diffraction (electronic supplementary material, figure S1). The natural halloysite shows characteristic sets of peaks that can be indexed as halloysite—10 Å (H10), anhydrous halloysite (H7) and natroalunite (N). In the patterns of HIHs, however, all these characteristic peaks have significantly decreased or disappeared, ascribed to the dehydration and thermal destruction of HNTs during the sample preparation [36,37]. A new peak appeared at 2*θ* = 44.6°, which can be indexed as the (110) plane of the body-centred cubic α-Fe (JCPDF no. 65-4899). With the increase of ageing time, the intensity of this peak decreased significantly, suggesting a declined crystallinity. In addition, a set of peaks gradually appeared with the increase of ageing time. It can be indexed as Fe_2_O_3_ (maghemite, JCPDF no. 39-1346). Its (311) peak appeared at 2*θ* = 35.6°, corresponding to the basal spacing of 0.26 nm that was observed in the TEM images. It is testified that the prepared iron nanoparticles have been partly oxidized. Furthermore, the remaining peak at 2*θ* = 26.6° may be ascribed to the (003) plane of carbon (JCPDF no. 26-1079) (produced from the carbonation of Fe^2+^-oleate complex) rather than the residual H10 phase. The XRD characterizations indicate that we have successfully prepared iron/halloysite nanocomposites.

**Figure 3.**
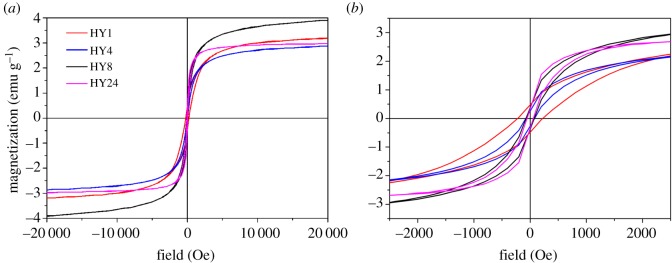
(*a*) Magnetization and (*b*) magnified magnetization curves of hollow iron/halloysite nanocomposites prepared with different ageing times.

Because the structure of iron nanoparticles changes with ageing time in this study, it is suggested that a variation of ageing time could change the physico-chemical properties of HIHs. As shown in figure 3, all samples show relatively low magnetizations due to their low loading of iron nanoparticles. With the ageing time increasing from 1 to 24 h, the saturation magnetization (*M*_s_) value generally kept unchanged at approximately 2.9–3.2 emu g^−1^, showing a maximum of 3.90 emu g^−1^ at an ageing time of 8 h. The remnant magnetization value and coercivity that directly related to the microwave performance of a material [38], however, declined significantly with ageing time. For instance, the coercivity of the samples decreased from 220 Oe at an ageing time of 1 h, to 75 Oe at 4 h, and further to 55 Oe at 24 h. This phenomenon may be ascribed to the decreased shape anisotropy with increasing proportion of solid iron nanoparticles [39,40]. Therefore, it is suggested the hollow iron nanoparticles should present higher magnetic loss for incident microwave than the solid nanoparticles.

The microwave absorption performance of the samples was further evaluated by studying their permeability and permittivity values (electronic supplementary material, figures S2 and S3). The real permittivity (*ε′*) values of HNTs are approximately 2.8 with a peak at frequency of 12 GHz. The prepared HIHs show slightly decreased *ε′* values of approximately 2.6. The imaginary permittivity (*ε′′*) values of HNTs fall in a range of 0.02–0.15, while that of HIHs ranged from 0.01 to 0.12. All samples present calculated dielectric loss tangents (tan *δ*_e_ = *ε′′*/*ε′*) of less than 0.06. They show almost identical positions of permittivity peaks, indicating that the magnetic iron nanoparticles contribute slightly for the dielectric loss of the nanocomposites. The HIHs present a tiny peak at frequency of 3.5 GHz, which may be caused by the eddy current effect of iron nanoparticles. The permeability curves of the samples are significantly changed by the loading of iron nanoparticles. All samples show real permeability (*μ′*) values of 1.0–1.1 with comparable positions of peaks. The HIHs, however, exhibited dramatically elevated imaginary permeability (*μ′′*) values and the corresponding magnetic loss (tan *δ*_m_ = *μ′′*/*μ′*) values at frequency of 10–15 GHz. According to the *C* value (*C* = *μ′′*(*μ′*)^−2^*f*^−1^) introduced by Wu *et al.* [41], the magnetic loss of both the HNTs and the HIHs is dominated by eddy current effect. HY1 and HY4 exhibit two different peaks at frequency of 11–14 GHz, indicating the presence of natural resonance [42]. The contribution of natural resonance was quite low and was limited to a narrow frequency range due to the low loading of iron nanoparticles. The HY1 and HY4 showed the highest *μ′′* and tan *δ*_m_ values, suggesting that the hollow iron nanoparticles present higher magnetic loss than the solid counterparts, attributed to their higher coercivity and remnant magnetization values.

**Figure 4.**
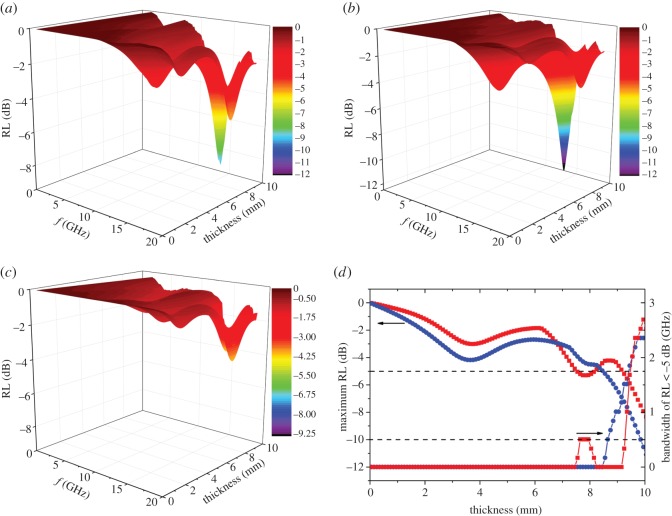
Variations of RL of microwaves by (*a*) natural halloysite (HNTs), (*b*) HY1 and (*c*) HY24 as functions of thickness and frequency. (*d*) Variations of maximum RL and bandwidth of RL < −5 dB achieved by HNTs (red squares) and HY1 (blue dots) as a function of thickness.

Based on the relative complex permittivity and permeability values of the samples, we calculated and compared their microwave absorption performance as functions of frequency and thickness. As shown in figure 4, both the HNTs and HIHs present relatively low microwave absorption at frequency of 2–18 GHz with reflection loss (RL) > −10 dB. In addition, the maximum bandwidth of RL < −5 dB appears at thicknesses of greater than 7 mm. When compared with HNTs, HY1 exhibits higher maximum RL values at thickness of 0–10 mm. Although the HNTs (2.7 GHz) show larger maximum bandwidth of RL < −5 dB than HY1 (2.4 GHz), the absorption range of HY1 of RL < −5 dB (8.5–10 mm) is wider than that of HNTs (9.2–10 mm). In addition, the maximum bandwidth of RL < −10 dB of HY1 achieved 0.3 GHz, while the maximum RL values of HNTs are all higher than −10 dB (electronic supplementary material, table S1). Therefore, the loading of hollow iron nanoparticles could enhance the microwave absorption performance of HNTs. On accounts of the magnetic and catalytic behaviours of iron nanoparticles, the prepared nanocomposites gain potential applications in organic wastewater treatment, with advantages of high adsorption, catalytic degradation and magnetic separation [25,43–45].

The microwave absorption performance of the nanocomposites was largely determined by the structure of the loaded iron nanoparticles. As shown in figure 4, electronic supplementary material, figure S4 and table S1, the RL values and absorption bandwidth of the samples vary with the ageing time. With the increasing ageing time from 1 to 24 h, the bandwidth of RL < −2.5 dB and RL < −5 dB decreased from 5.9 and 2.4 GHz to 2.9 and 0 GHz, respectively. The maximum RL also declined from −10.5 to −4.2 dB. Because the proportion of hollow iron nanoparticles decreases with ageing time, it is suggested that the hollow iron nanoparticles present higher microwave absorption than the solid ones. This phenomenon is attributed to the higher magnetic loss of hollow iron nanoparticles caused by their enhanced coercivity and remnant magnetization values. Nevertheless, the microwave absorption performance of HIHs is relatively limited when compared with other halloysite nanocomposites (electronic supplementary material, table S1) [2,46], possibly ascribed to the low loading of hollow iron nanoparticles in this study. In addition, the performance of HIHs is also quite poor compared with traditional microwave absorbers, such as carbon nanotubes, Fe and Co [24,47,48]. We will try to increase the loading of iron nanoparticles and enhance the magnetic and electromagnetic performances of HIHs in our following study.

## 3. Conclusion

In summary, we successfully prepared a novel halloysite nanocomposite with hollow iron nanoparticles loaded on the surface of HNTs. The structure of the iron nanoparticles could be controlled by the ageing time during preparation. With the increasing of ageing time, the proportion of hollow iron nanoparticles decreases, while that of solid nanoparticles increases. The hollow iron nanoparticles showed much higher remnant magnetization and coercivity values than the solid ones, and accordingly exhibited higher microwave absorption performance. The maximum RL values and bandwidth of RL < −5 dB of the nanocomposite achieved −10.5 dB and 2.4 GHz, respectively. Nevertheless, the microwave absorption performance of the prepared nanocomposites was relatively low when compared with traditional electromagnetic wave absorbers. With advantages of magnetic separation, catalytic degradation capacity of iron nanoparticles and large adsorption volume of HNTs, the prepared HIH nanocomposites show potential applications in organic wastewater treatment. In addition, this study opens a new pathway to fabricate magnetic halloysite nanocomposites as microwave absorbers.

## Supplementary Material

Electronic Supplementary Information

## Data accessibility

The material and methods, figures and a table supporting this article have been uploaded as part of the electronic supplementary material.
