# Supplementary material for: Preparation of hollow iron/halloysite nanocomposites with enhanced electromagnetic performances
Source: R Soc Open Sci. 2018 Jan 24;5(1):171657. doi: 10.1098/rsos.171657 (PMC5792942; doi:10.1098/rsos.171657)
Supplement: Electronic Supplementary Information [file rsos171657supp1.docx]

† Electronic Supplementary Information (ESI) available:

**1 Materials and methods**

*1.1 Materials*

Anhydrous ethanol, hexane, and ferrous chloride were purchased from Sinopharm chemical Reagent Co., Ltd (Shanghai, China). Sodium oleate was obtained from Aladdin Industrial Corporation (Shanghai, China). Halloysite was supplied by Zhongwei mineral materials Corporation (Xingtai, China). All materials were used as received without further purification.

*1.2 Preparation of hollow-Fe/halloysite nanocomposites*

The hollow-Fe/halloysite nanocomposites (HIH) were synthesized by a “oleate” method. In a typical procedure, 80 mmol FeCl_2_, and 160 mmol sodium oleate were added into mixed solvents of 100 mL H_2_O, 100 mL ethanol, and 200 mL hexane. The mixture was heated and refluxed at 70 ^o^C for 4 h. The obtained Fe^2+^-oleate complex was extracted and washed throughly by distrilled water in a separatory funnel. The product was dryed at 70 ^o^C to remove the residual ethanol and hexane, and then 110 ^o^C to remove water.

Then the Fe^2+^-oleate complex was mixed with natural halloysite at a mass ratio of 1:1. After aging for a given time, the mixture was first calcinated at 450 ^o^C under N_2_ atmosphere for 2 h and then switched to N_2_+H_2_ atmosphere for 8 h. The obtained hollow-Fe/halloysite nanocomposite was named as HY*t* according to the aging time (*t*). For instance, HY1 represents an aging time of 1 h.

*1.3 Characterizations*

The crystal structure of the samples were examined by a D/max 2550 X-ray diffractometer (XRD) (Riguaku, Japan) with Cu Kα radiation (λ=0.15406 nm) from 3^o^ to 75^o^. The operation voltage and current were maintained at 40 kV and 34 mA, respectively. The micro-morphologies of the samples were studied by a JEM-2010 transmission electron microscope (TEM) (JEOL, Japan) at an accelerating voltage of 200 kV. The magnetic measurements were performed by an MPMS-XL-5 SQUID magnetometer (Quantum Design, USA) at room temperature with magnetic field ranging from -20000 Oe to 20000 Oe.

The relative complex permittivity and permeability of the samples were determined by a HP8720ES vector network analyzer (Agilent, USA) using T/R coaxial line method. The measurements were conducted at a microwave frequency of 8-18 GHz and a thickness of 2 mm. The volume fractions of the samples were 30%. The relative complex permittivity (*ε*=*ε*'-*jε*'') and permeability (*μ*=*μ*'-*jμ*'') were obtained from the measured T/R coefficients. The reflection loss (RL) values of the absorbers were calculated from the measured complex permittivity and permeability values using the following equations:

$Z_{in}=Z_{0}\sqrt{\frac{\mu}{\varepsilon}}\tanh[j\frac{2\pi fd}{c}\sqrt{\mu\varepsilon}]$ (1)

$RL(dB)=20\log\left|\frac{Z_{in}-Z_{0}}{Z_{in}+Z_{0}}\right|$ (2)

where Z_in_ and Z_0_ are the impedance of the absorber and the air, respectively. *ε* and *μ* are the complex permittivity and permeability of the absorber, respectively. *f* is the frequency of electromagnetic wave. *d* is the thickness of the absorber. *c* is the velocity of light.

**2 Supporting figures and tables**


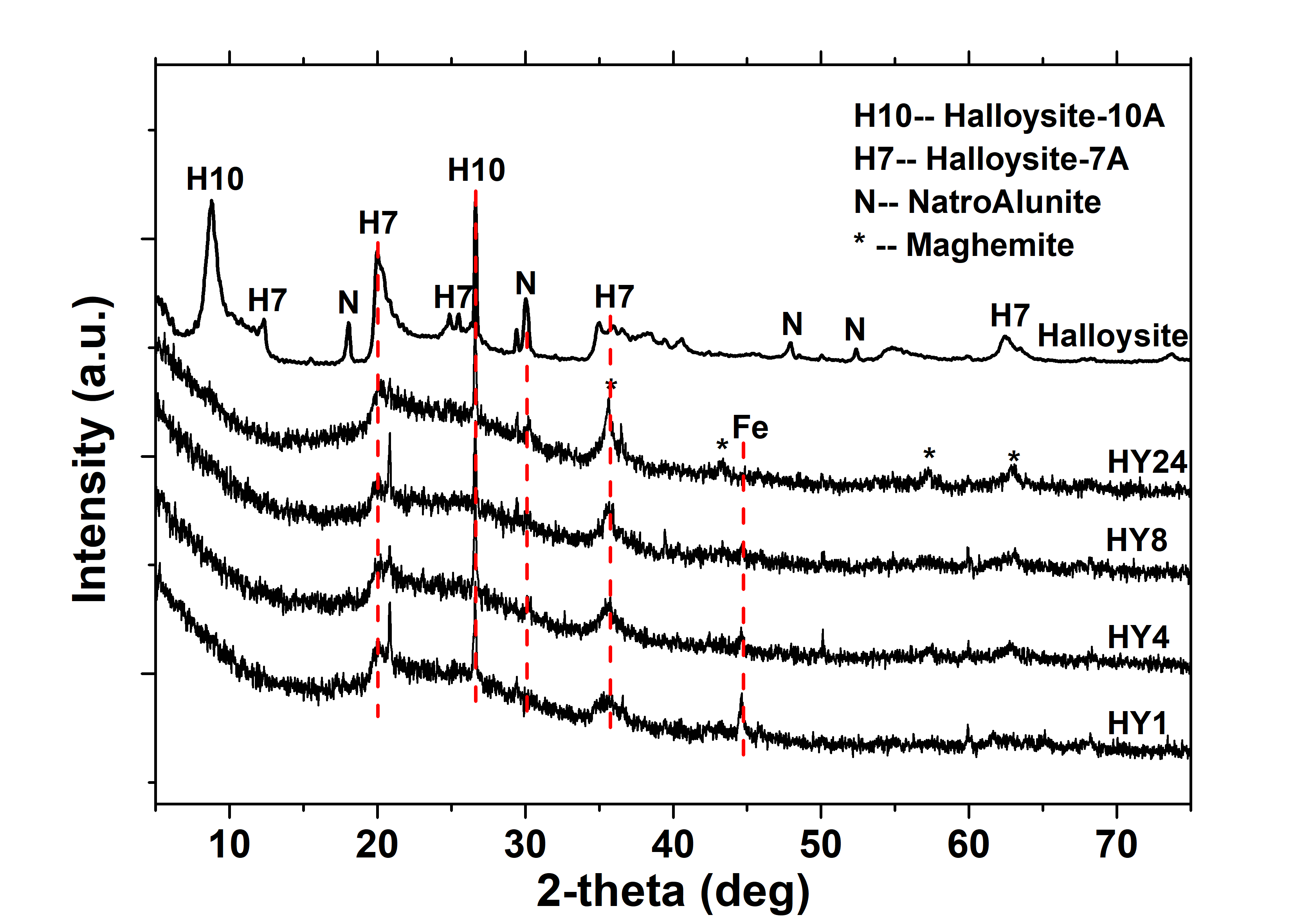


**Fig. S1** XRD patterns of natural halloysite (HNTs) and hollow iron/nanocomposites prepared at different aging times.


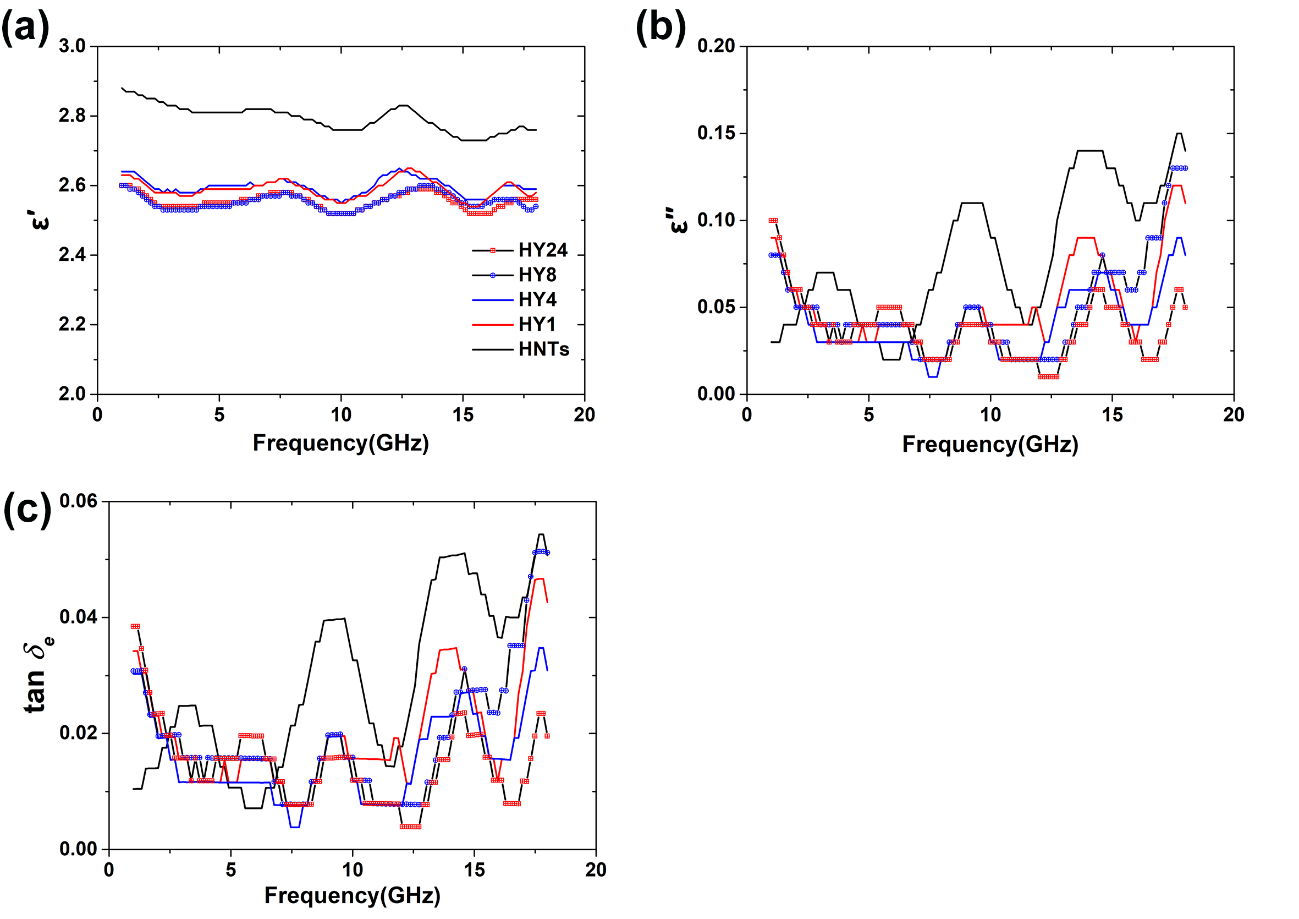


**Fig. S2** Frequency dependence of (a) relative real permittivity (*ε′*), (b) imaginary permittivity (*ε″*), and (c) dielectric loss tangents (tan *δ_e_* = *ε″*/ *ε′*) of natural halloysite (HNTs) and hollow iron/halloysite nanocomposites prepared at different aging times.


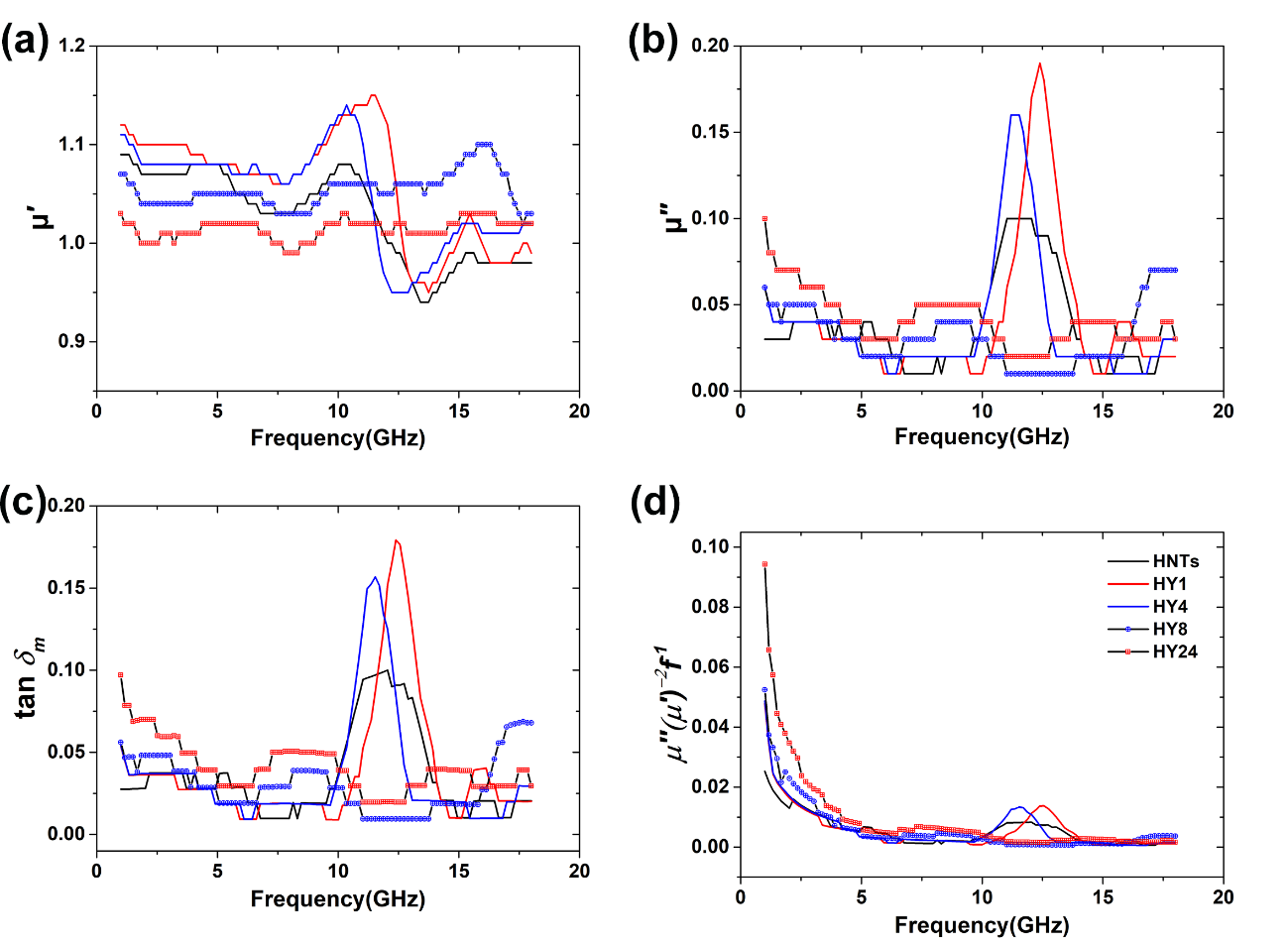


**Fig. S3** Frequency dependence of (a) relative real permeability (*μ*'), (b) imaginary permeability (*μ*''), (c) magnetic loss tangents (tan *δ_m_* =*μ″*/*μ′*), and (d) C value (C=*μ*'' (*μ*')^-2^*f*^-1^) of natural halloysite (HNTs) and hollow iron/halloysite nanocomposites prepared at different aging times.


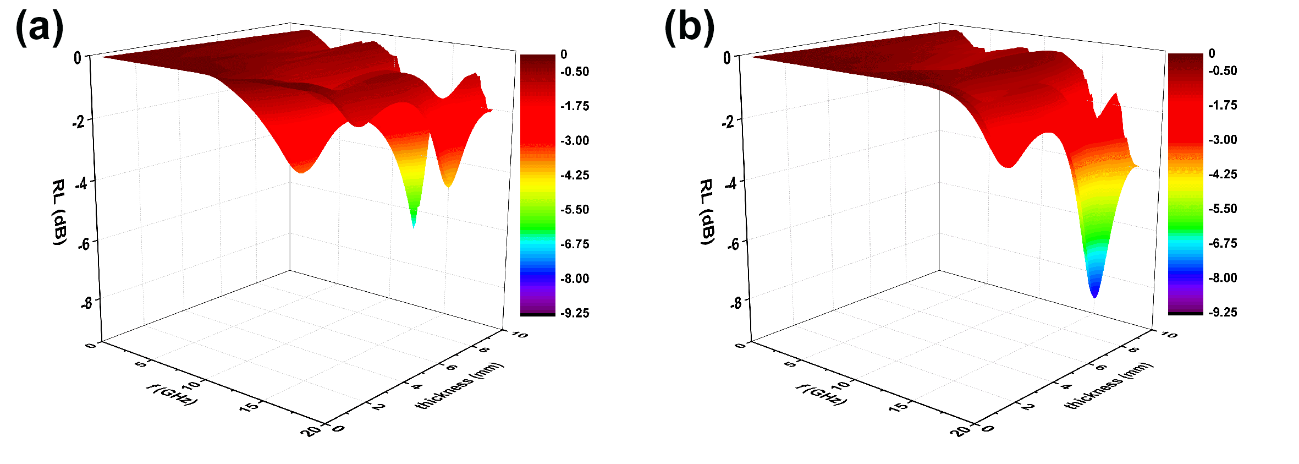


**Fig. S4** Variations of reflection loss (RL) of microwaves by hollow iron/halloysite nanocomposites as functions of frequency and thickness. (a) HY4: sample prepared at an aging time of 4 h; (b) HY8: aging time of 8 h.

Table S1 Comparisons on the magnetic and microwave absorption performance of natural halloysite (HNTs), the hollow iron/halloysite nanocomposites, other halloysite nanocomposites, and traditional microwave absorbers.

| Sample | Saturation magnetization (emu g^-1^) | Remnant magnetization (emu g^-1^) | Coercivity (Oe) | Bandwidth of RL<-2.5 (GHz) | Bandwidth of RL<-5 (GHz) | Bandwidth of RL<-10 (GHz) | Maximum RL (dB) | Reference |
| --- | --- | --- | --- | --- | --- | --- | --- | --- |
| HNTs |  |  |  | 7.1 | 2.7 | 0 | -7.9 | This study |
| HY1 | 3.19 | 0.48 | 220 | 5.9 | 2.4 | 0.3 | -10.5 |  |
| HY4 | 2.88 | 0.26 | 75 | 3.9 | 0.7 | 0 | -5.6 |  |
| HY8 | 3.90 | 0.38 | 70 | 3.9 | 1.7 | 0 | -8.1 |  |
| HY24 | 2.98 | 0.33 | 55 | 2.9 | 0 | 0 | -4.2 |  |
| Encapsulated MnFe_2_O_4_+halloysite | 4.21 | 0.26 | 40 |  | 6.12 | 4.08 | -36 | (1) |
| MnFe_2_O_4_+halloysite | 1.09 | 0.12 | 20 |  | 0 | 0 |  |  |
| PEDOT/PSS/halloysite |  |  |  |  |  | 2.0 | -16.3 | (2) |
| Carbon nanotubes |  |  |  |  |  | 5.8 | -25.02 | (3) |
| Hollow Co |  |  |  |  |  | 4.8 | -47.3 | (4) |
| Porous Fe |  |  |  |  | 6.8 | 3.0 | -21.86 | (5) |

**References:**

1. A.-B. Zhang, S.-T. Liu, K.-K. Yan, Y. Ye and X.-G. Chen. 2014. Facile preparation of MnFe_2_O_4_/halloysite nanotubular encapsulates with enhanced magnetic and electromagnetic performances. *RSC Adv* **4**, 13565 - 13568.

2. S.-J. Luo, P. Zhang, Y.-A. Mei, J.-B. Chang and H. Yan. 2016. Electromagnetic interference shielding properties of PEDOT/PSS–halloysite nanotube (HNTs) hybrid films. *J Appl Polym Sci* **133**, 44242.

3. T. Zhao, C. Hou, H. Zhang, R. Zhu, S. She, J. Wang, T. Li, Z. Liu and B. Wei. 2014. Electromagnetic Wave Absorbing Properties of Amorphous Carbon Nanotubes. *Sci Rep* **4**, 5619.

4. P. Yang, X. Zhao, Y. Liu and Y. Gu. 2017. Facile, Large-Scale, and Expeditious Synthesis of Hollow Co and Co@Fe Nanostructures: Application for Electromagnetic Wave Absorption. *J Phys Chem C* **121**, 8557-8568.

5. X.-G. Chen, J.-P. Cheng, S.-S. Lv, P.-P. Zhang, S.-T. Liu and Y. Ye. 2012. Preparation of porous magnetic nanocomposites using corncob powders as template and their applications for electromagnetic wave absorption. *Compos Sci Technol* **72**, 908-914.
